# How often do Australian government-funded interventional trials exclude adults with intellectual disability? A retrospective registry analysis of ANZCTR records, 2022–2024

**DOI:** 10.1136/bmjopen-2026-119053

**Published:** 2026-10-20

**Authors:** Andrea Kargodorian, Rebecca Christidis, Jenny Downs, Helen Leonard, Julian Trollor

**Affiliations:** 1 National Centre of Excellence in Intellectual Disability Health, UNSW Sydney, UNSW Medicine & Health, Sydney, New South Wales, Australia; 2 The Kids Research Institute Australia, Nedlands, Western Australia, Australia

**Keywords:** Clinical Trial, Health Equity, MEDICAL ETHICS, STATISTICS & RESEARCH METHODS, Vulnerable Populations

## Abstract

**Abstract:**

**Objective:**

To quantify the frequency and types of direct and indirect exclusion criteria affecting adults with intellectual disability in Australian government-funded interventional clinical trials and to examine variation in exclusion patterns by trial characteristics.

**Design:**

Retrospective analysis of trial records registered on the Australian New Zealand Clinical Trials Registry (ANZCTR).

**Data sources:**

All ANZCTR records for interventional trials funded by an Australian government source and registered between 1 January 2022 and 31 December 2024 were downloaded from the ANZCTR website. Eligibility criteria were extracted from free-text registry fields. Exclusion criteria were coded using an adapted framework distinguishing direct exclusions (ie, diagnosis, consent capacity, cognitive capacity) and indirect exclusions (ie, language, technology requirements, staff discretion).

**Study selection:**

Trials were excluded if they were phase 0, 1, 1/2 or 2, if the minimum participant age was >55 years or if the participant maximum age was <18 years. Of 857 records screened, 685 trials met inclusion criteria.

**Results:**

Over half of trials (53%) contained no direct or indirect exclusion codes. One-third (34.2%) contained at least one direct exclusion criterion, most commonly requirements related to consent capacity (17.2%). Exclusion based on diagnostic labels was seen in 13.1% of trials and 4.8% explicitly excluded participants with intellectual disability. Indirect exclusion criteria were present in 25% of trials, including English-language requirements (12.3%) and staff-discretion codes (9.2%). Patterns varied by health condition and intervention type: cancer and drug trials showed the highest overall exclusion rates, while large trials (≥400 participants) were least likely to have any exclusions but more likely than small trials to explicitly exclude intellectual disability.

**Conclusions:**

A substantial minority of Australian government-funded trials potentially exclude people with intellectual disability through diagnostic labels, assumptions about capacity and indirect mechanisms. Trials excluding potential participants on the basis of unassessed cognitive or consent capacity are inconsistent with ethics guidance about individualised assessment and supported decision-making. Findings suggest significant room for improvement in eligibility design, such as ensuring justification for diagnostic and capacity-based exclusions, and the adoption of validated approaches to supporting participation of adults with intellectual disability.

**STRENGTHS AND LIMITATIONS OF THIS STUDY**

- Analysed a large sample of Australian government-funded interventional clinical trials using data from the Australian New Zealand Clinical Trials Registry.
- Applied an established coding framework to systematically identify both direct and indirect exclusion criteria from free-text eligibility fields.
- Independent double coding of exclusion criteria strengthened the reliability of data extraction and classification.
- The study used publicly available registry records, which limited analysis to the eligibility information reported in those records.
- Data and findings cannot be generalised to children or older adults with intellectual disability.

## Introduction

 Clinical trials are central to evidence-based medicine, providing information needed to evaluate the safety and effectiveness of medicines, devices, services and interventions, and informing treatment standards, clinical practice and health policy.^1^ Their value depends on whether trial populations reflect the diversity of people who use resulting interventions. When marginalised groups are excluded or under-represented, the validity of findings narrows and inequities in care can widen.^2–4^ In Australia, approximately 2% of the population live with an intellectual disability,^5^ and this group experiences health disparities, including higher rates of chronic disease, avoidable hospitalisation and premature mortality.^6 7^ Exclusion from clinical trials limits understanding of treatment effects for this population and perpetuates structural disadvantage.^8^

Australia has a clinical trials landscape with more than 18 000 trials and approximately 8.7 million participants in trials registered between 2006 and 2020.^9^ Trials remain strategically important with Australia’s draft National Health and Medical Research Strategy 2026–2036 which positions clinical trials as central to achieving equitable access to innovative interventions, calls for improved access across all demographic groups and specifies that equitable research translation ensures no one is left behind.^10^ The Medical Research Future Fund (MRFF) identifies equitable outcomes from clinical trials as a key priority,^11^ with recent reports indicating more than 744 clinical trials have been supported by MRFF funding.^12^ International evidence shows that not all population groups have benefited equally from biomedical research, and recognising these inequities is a key first step towards correcting them.^13^

Exclusion of people with intellectual disability from clinical trials is based on direct or indirect criteria that cumulatively limit participation. Across interventions, approximately 20%–85% of trials contain at least one exclusion criterion that disproportionately affects underserved groups,^3 4^ with even higher rates reported in oncology and critical care trials.^14–20^ One review found that almost two-thirds (61.4%) of interventional trials between 2010 and 2020 explicitly excluded people with cognitive impairment, and many exclusions could have been avoided through minor design adjustments.^4^ Across studies, exclusion criteria are frequently applied without clear justification, particularly when related to cognitive or consent capacity.^3 16 21^ Exclusion based on diagnostic labels is also present, with broad psychiatric or behavioural criteria found in two-thirds of trials examined in recent studies.^22 23^ Indirect exclusion occurs when participation relies on tasks that people with intellectual disability may find challenging, such as self-report, online platforms or reading English fluently; about one in five Australian trials exclude participants with limited English proficiency.^14^ Indirect barriers can arise when eligibility is determined by the ‘opinion of the investigator’, creating scope for subjective decision-making.^17 24^ When considered together, these direct and indirect exclusions can significantly limit opportunities to participate in clinical trials for those with cognitive or communication barriers.

While international work has mapped direct and indirect exclusion patterns using large registries such as ClinicalTrials.gov and the EU Clinical Trials Register, little is known about how exclusion patterns appear within Australia’s clinical trials landscape.^13 16^ No study has examined how often direct and indirect exclusions are represented in interventional trials funded by the Australian government. Understanding these patterns is essential to improving inclusive practice and ensuring that publicly funded research benefits all Australians. This study aims to explore the extent and nature of both direct and indirect exclusion of adults with intellectual disability in Australian government-funded interventional clinical trials. Specifically, it sought to quantify the frequency of direct and indirect exclusion criteria; describe the types of exclusions most commonly used; and explore how exclusion patterns vary by health condition, intervention type and trial size, using data from the Australian New Zealand Clinical Trials Registry (ANZCTR) between 2022 and 2024.

## Methods

### Data source and search

We identified clinical trial records using the advanced search function on the ANZCTR (accessed at: https://www.anzctr.org.au/TrialSearch.aspx), which uses WHO-compliant definitions for interventional studies. We chose the ANZCTR for this search as other registries lack structured data around Australian government-funded trials. The National Health and Medical Research Council (NHMRC) guidance states that clinical trials must be registered with ANZCTR prior to recruitment.^25^ The search was limited to interventional trials with a start date between 1 January 2022 and 31 December 2024, were open to recruitment or had completed recruitment (ie, not yet recruiting, recruiting, active not recruiting or completed), were recruiting in Australia, and the trial was funded by the government. We extracted the data as an Excel file that contained full ANZCTR trial records on 7 June 2025. We extracted full trial records. (For full data structure, see ANZCTR data field definitions available at: https://www.anzctr.org.au/docs/ANZCTRDatafieldexplanation.pdf.) We retained the following fields: trial ID, ACTRN, minimum age, minimum age type, maximum age, maximum age type, exclusion criteria, target sample size, recruitment state, study type, trial purpose phase, condition code, intervention code, funding source and primary sponsor type.

### Eligibility criteria

We excluded early-phase trials (phase 0, 1 and 1/2) due to their emphasis on safety and pharmacokinetics, where exclusion of vulnerable populations is potentially justifiable. Trials with a minimum age of 55 years were excluded to reduce confounding related to age-related cognitive decline. Trials with a maximum age of 18 years were also excluded.

### Data extraction and coding

We recorded the funding source name for each trial record as a free-text field and categorised it as NHMRC, MRFF and other government sources. For direct and indirect exclusion codes, we applied an existing coding framework.^18^ We coded direct exclusion when trial exclusion criteria referenced cognitive capacity (eg, ‘Mini-Cog score <4’, ‘DSM-5 mini assessment’, ‘Mini-Mental State Examination’, ‘Short Portable Mental Status Questionnaire’, ‘Montreal Cognitive Assessment score <10’), cognitive capacity to engage in study procedures (eg, ‘unable to follow instructions’) or consent capacity (eg, ‘unable to provide informed consent’). We coded whether cognitive or consent capacity assessments were specified. Direct exclusion was also coded when trial exclusion criteria referenced diagnostic labels of developmental disability, intellectual disability or other (eg, diagnosed psychiatric condition as defined by DSM-V criteria). We coded indirect exclusion when exclusion criteria referenced language proficiency (eg, ‘must read and write in English’), access to technology (eg, ‘must have smartphone or internet access’), ability to use technology (eg, ‘limitations to the use of mobile technology’, ‘digital illiteracy’, ‘unable to participate in e-health components’) or research staff discretion (eg, ‘in the opinion of the investigator’). Subcategories of research staff discretion were coded, including health status (eg, ‘have any health condition or illness that, in the opinion of the participant or researcher’, ‘any condition that, in the judgement of the investigator’, ‘any other illnesses as assessed by the investigator’), safety concerns (eg, ‘any other condition that would, in the investigator’s judgement, contraindicate the patient’s participation in the clinical study due to safety concerns’, ‘in the opinion of the investigator, may deem it unsafe to participate’, ‘any clinical contraindication that precludes safe completion of the programme in the judgement of the project team’), potential to confound findings and other unspecified reasons (eg, ‘might confound the interpretation of the study results’, ‘or the feasibility of achieving the study objectives’, ‘considered by the clinical investigator (CI) to be unsuitable for participation in the study’). All included trial records were coded independently by two authors (AK and RC) as present or absent using NVivo software. We conducted keyword searches using a five-word context window to capture relevant phrasing. All coding across each trial record was then reviewed in full by both authors and any discrepancies in coding were resolved through detailed discussion until consensus was reached.

We categorised trial records by intervention type, health condition, age group and trial phase. We summarised the frequency of inclusion and exclusion criteria across these categories and used descriptive statistics to characterise the prevalence of each exclusion type. Trial records were grouped by target sample size to examine patterns across different trial scales (small <100, medium 100–399, large >400). We compared exclusionary criteria between drug and prevention trials to explore whether intervention type influences the application of exclusion criteria.

### Statistical analysis

Descriptive statistics were used to summarise the frequency and distribution of direct and indirect exclusion criteria across trial characteristics. Results are reported as counts and percentages. We did not perform inferential statistical testing. We used a descriptive approach because the study aimed to summarise registry data and subgroup comparisons were exploratory rather than hypothesis-driven.

## Results

### Identification and inclusion of eligible trials

As shown in figure 1, 857 trial records were identified on anzctr.org.au. Consistent with other studies,^16 18 22^ we excluded early phase trials where the exclusion of potentially vulnerable groups may be more justifiable (phase 0, n=5; phase 1, n=17; phase 1/2, n=3; phase 2, n=42), studies with a minimum age ≥55 years^18^ to reduce confounds when interpreting results (ie, trials related to ageing; n=48) and trials with only child participants (maximum age <18 years, n=57) were excluded because the audit focused on adult participation in clinical trials; studies with both child and adult participants were retained. In total, 172 trials were excluded, which left 685 trial records for coding and analysis.

**Figure 1:**
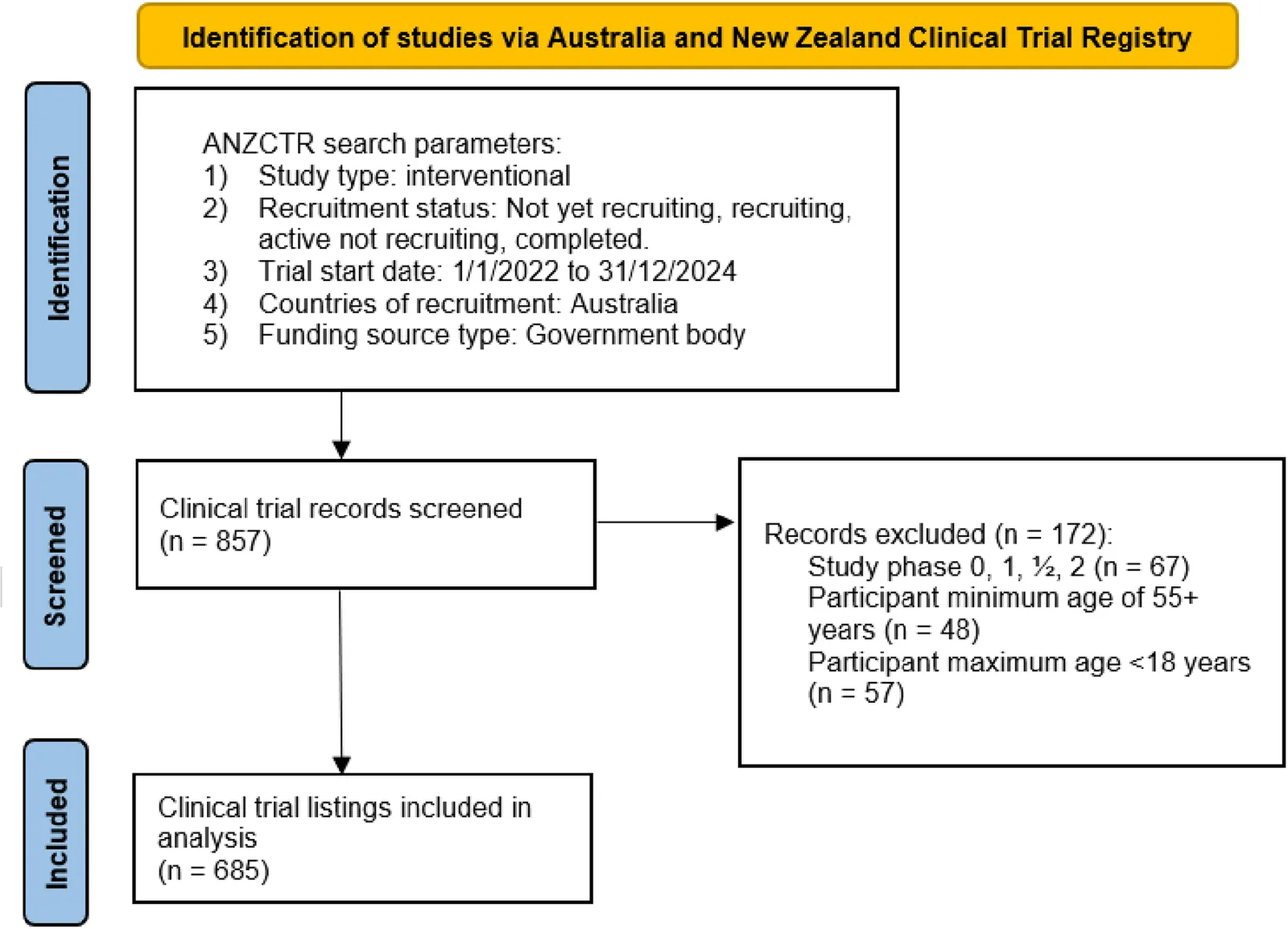
Screening of clinical trials via ANZCTR search. ANZCTR, Australian New Zealand Clinical Trials Registry.

### General characteristics

Trial characteristics are summarised in table 1. Trials represented all intervention and condition categories and were mostly recruiting or not yet recruiting. University sponsors were most common (58%), most trials set a minimum age of ≥18 years (74.9%), and treatment-drug trials comprised a minority (10.8%).

**Table 1:** Characteristics of included clinical trials

Characteristic	n (%)
Trial phase	
Not applicable	619 (90.4)
Phase 2/phase 3	10 (1.5)
Phase 3	27 (3.9)
Phase 3/phase 4	5 (0.7%)
Phase 4	24 (3.5)
Trial intervention*	
Behaviour	238 (34.7)
Diagnosis/prognosis	45 (6.6)
Early detection/screening	57 (8.3)
Lifestyle	96 (14)
Other interventions	2 (0.3)
Prevention	121 (17.7)
Rehabilitation	96 (10.1)
Treatment: devices	87 (12.7)
Treatment: drugs	76 (11.1)
Treatment: other	232 (33.9)
Treatment: surgery	12 (1.8)
Health condition category*	
Alternative and complementary medicine	3 (0.4)
Anaesthesiology	14 (2)
Blood	2 (0.3)
Cancer	90 (13.1)
Cardiovascular	73 (10.7)
Diet and nutrition	55 (8)
Ear	3 (0.4)
Emergency medicine	11 (1.6)
Eye	6 (0.9)
Human genetics and inherited disorders	5 (0.7)
Infection	31 (4.5)
Inflammatory and immune system	18 (2.6)
Injuries and accidents	38 (5.6)
Mental health	183 (26.7)
Metabolic and endocrine	55 (8)
Musculoskeletal	59 (8.6)
Neurological	93 (13.6)
Oral and gastrointestinal	33 (4.8)
Other	3 (0.4)
Physical medicine/rehabilitation	53 (7.7)
Public health	179 (26.1)
Renal and urogenital	15 (2.2)
Reproductive health and childbirth	52 (7.6)
Respiratory	38 (5.6)
Skin	7 (1)
Stroke	24 (3.5)
Surgery	20 (2.9)
Recruitment status	
Not yet recruiting	273 (39.9)
Active, not recruiting	39 (5.7)
Recruiting	284 (41.5)
Completed	89 (13)
Trial size	
Small (target trial size <100)	241 (35.2)
Medium (target trial size 100–399)	239 (34.9)
Large (target trial size >400)	205 (30)
Government funder*	
National Health and Medical Research Council (NHMRC)	323 (47.2)
Medical Research Future Fund (MRFF)	153 (22.3)
Other	284 (41.5)
Primary sponsor type	
Charities/societies/foundations	15 (2.2)
Commercial sector/industry	13 (1.9)
Government body	79 (11.5)
Hospital	63 (9.2)
Individual	47 (6.9)
Other	39 (5.7)
Other collaborative groups	25 (3.6)
University	404 (59)
Minimum age	
<18 years	135 (25.1)
>18 years <55 years	550 (74.9)
Total number of trials	685

* Multiple selections were allowed within a single clinical trial record, and percentages may not equal 100%.

### Overall exclusion rates

Table 2 shows exclusion criteria. Overall, 53.2% of trials had no exclusion codes. Just over a third included direct exclusion codes (34.2%), most related to consent capacity (17.2%) and diagnosis (13.0%). Exclusions were often applied without formal assessment across consent capacity (17.2%), cognitive capacity (7.8%) and cognitive capacity to engage in study procedures (3.9%). Among exclusions based on diagnostic labels, the most frequent were coded as ‘other’ (8.4%) followed by explicit exclusion of people with intellectual disability (4.8%). Indirect exclusion was present in 25.0% of trials, most commonly English language proficiency (12.1%) and research staff discretion codes (9.2%). Technology-related exclusion was less frequent (access 4.5%; ability to use 1.3%). Within research staff discretion codes, the most common were health (3.9%), other unspecified reasons (3.6%) and safety (2.7%).

**Table 2:** Clinical trials with direct and indirect exclusion criteria

	n (%)
**Direct exclusion**	234 (34.2)
Consent capacity (any code)	119 (17.4)
Assessment	1 (0.1)
No assessment	119 (17.4)
Cognitive capacity (any code)	69 (10.1)
Assessment	15 (2.2)
No assessment	54 (7.9)
Cognitive capacity to engage in study procedures (any code)	28 (4.1)
Assessment	2 (0.3)
No assessment	27 (3.9)
Diagnosis	90 (13.1)
Developmental disability	7 (1.0)
Intellectual disability	33 (4.8)
Other	58 (8.5)
**Indirect exclusion**	171 (25.0)
Access to technology	30 (4.4)
Ability to use technology	9 (1.3)
Ability to read and write in English	84 (12.3)
Research staff discretion (any code)	63 (9.2)
Ability to complete study procedures	13 (1.9)
Health	27 (3.9)
Potential to confound findings	14 (2.0)
Safety	19 (2.8)
Other	24 (3.5)
**No exclusion codes**	363 (53.0)
Total number of trials	685

### Exclusion rates by health condition

Exclusion criteria by trial health condition category are shown in table 3. Across the three most common health condition categories, mental health, public health and cancer, patterns of exclusion varied. Cancer trials had the lowest proportion with no exclusion codes (35.2%), compared with mental health (55.7%) and public health (58.9%). Direct exclusion was highest in cancer trials (47.3%), followed by mental health (33.3%) and public health (31.1%). Consent capacity exclusion was most frequent in cancer trials (28.6%) and was without assessment. In contrast, consent capacity exclusion was least frequent in mental health trials (13.1%). Diagnostic-label exclusions were most common in mental health trials (18.0%), including explicit exclusion of people with intellectual disability (8.2%). Cancer trials had a diagnosis-based exclusion rate of 13.2% and these were all other diagnoses beyond developmental or intellectual disability (eg, diagnosed psychiatric condition as defined by DSM-V criteria). Indirect exclusion was also highest in cancer trials (36.3%), particularly staff discretion (22.0%) and English language proficiency (8.8%). Public health trials had the lowest rates of indirect exclusion (21.1%), though still included criteria related to technology access (6.7%) and staff discretion (6.7%). Mental health trials showed a broader spread of indirect exclusion codes, with 14.8% requiring English proficiency, 7.7% requiring access to technology and 4.9% with any research staff discretion.

**Table 3:** Clinical trials by health condition category*

	Mental health	Public health	Cancer
	**n (%**)	**n (%**)	**n (%**)
**Direct exclusion**	61 (33.3)	56 (31.3)	43 (47.8)
Consent capacity (any code)	24 (13.1)	34 (19.0)	26 (28.9)
Assessment	1 (0.5)	0 (0.0)	0 (0.0)
No assessment	24 (13.1)	34 (19.0)	26 (28.9)
Cognitive capacity (any code)	13 (7.1)	17 (9.5)	13 (14.4)
Assessment	5 (2.7)	3 (1.7)	3 (3.3)
No assessment	8 (4.4)	14 (7.8)	10 (11.1)
Cognitive capacity to engage in study procedures (any code)	9 (4.9)	6 (3.4)	6 (6.7)
Assessment	1 (0.5)	0 (0.0)	0 (0.0)
No assessment	9 (4.9)	6 (3.4)	6 (6.7)
Diagnosis	33 (18.0)	22 (12.3)	12 (13.3)
Developmental disability	3 (1.6)	2 (1.1)	0 (0.0)
Intellectual disability	15 (8.2)	13 (7.3)	0 (0.0)
Other	16 (8.7)	10 (5.6)	12 (13.3)
**Indirect exclusion**	46 (25.1)	38 (21.2)	32 (35.6)
Access to technology	14 (7.7)	12 (6.7)	5 (5.6)
Ability to use technology	1 (0.5)	3 (1.7)	1 (1.1)
Ability to read and write in English	27 (14.8)	13 (7.3)	8 (8.9)
Research staff discretion (any code)	9 (4.9)	12 (6.7)	19 (21.1)
Ability to complete study procedures	1 (0.5)	0 (0.0)	1 (1.1)
Health	4 (2.2)	2 (1.1)	4 (4.4)
Potential to confound findings	1 (0.5)	0 (0.0)	2 (2.2)
Safety	3 (1.6)	3 (1.7)	5 (5.6)
Other	3 (1.6)	7 (3.9)	12 (13.3)
**No exclusion codes**	102 (55.7)	105 (58.7)	32 (35.6)
Total number of trials	183	179	90

* Multiple selections were allowed within a single clinical trial record, and trials may be represented in more than 1 health condition category.

### Exclusion rates by intervention type

Exclusion criteria by trial intervention are shown in table 4. Direct exclusion is more common in drug trials (41.6%) than prevention trials (32.8%). Consent capacity exclusions were higher in drug trials (24.7%) than prevention (16.4%) and were almost always applied without assessment. Diagnostic-label exclusions were more prevalent in drug trials (18.2%), including explicit exclusion of intellectual disability (2.6%). Exclusion by cognitive capacity was more common in prevention trials (14.8%) than drug trials (6.5%). Prevention trials excluded intellectual disability more often (5.7%) but had fewer overall diagnostic exclusions (12.3%). Indirect exclusion was present in 33.8% of drug trials and 23.8% of prevention trials, with drug trials showing higher rates of staff discretion (26.0% vs 8.2%). Staff discretion codes in drug trials were highest for other unspecified reasons (13.0%) and health (11.7%). Technology-related exclusions were more common in prevention trials, with 8.2% requiring access to technology and 0.0% in drug trials. Overall, drug trials had a lower proportion of trials with no exclusion codes (39.0%) compared with prevention trials (53.3%).

**Table 4:** Clinical trials by target sample size

	Small	Medium	Large
	**n (%**)	**n (%**)	**n (%**)
**Direct exclusion**	84 (34.9)	87 (36.4)	63 (30.7)
Consent capacity (any code)	38 (15.8)	41 (17.2)	40 (19.5)
Assessment	1 (0.4)	0 (0.0)	0 (0.0)
No assessment	38 (15.8)	41 (17.2)	40 (19.5)
Cognitive capacity (any code)	30 (12.4)	27 (11.3)	12 (5.9)
Assessment	5 (2.1)	7 (2.9)	3 (1.5)
No assessment	25 (10.4)	20 (8.4)	9 (4.4)
Cognitive capacity to engage in study procedures (any code)	14 (5.8)	7 (2.9)	7 (3.4)
Assessment	0 (0.0)	2 (0.8)	0 (0.0)
No assessment	14 (5.8)	6 (2.5)	7 (3.4)
Diagnosis	37 (15.4)	28 (11.7)	25 (12.2)
Developmental disability	0 (0.0)	3 (1.3)	4 (2.0)
Intellectual disability	13 (5.4)	6 (2.5)	14 (6.8)
Other	26 (10.8)	20 (8.4)	12 (5.9)
**Indirect exclusion**	49 (20.3)	79 (33.1)	43 (21.0)
Access to technology	4 (1.7)	15 (6.3)	11 (5.4)
Ability to use technology	2 (0.8)	6 (2.5)	1 (0.5)
Ability to read and write in English	16 (6.6)	47 (19.7)	21 (10.2)
Research staff discretion (any code)	28 (11.6)	19 (7.9)	16 (7.8)
Ability to complete study procedures	9 (3.7)	3 (1.3)	1 (0.5)
Health	10 (4.1)	10 (4.2)	7 (3.4)
Potential to confound findings	7 (2.9)	3 (1.3)	4 (2.0)
Safety	9 (3.7)	7 (2.9)	3 (1.5)
Other	8 (3.3)	8 (3.3)	8 (3.9)
**No exclusion codes**	132 (54.8)	109 (45.6)	122 (59.5)
Total number of trials	241	239	205

### Exclusion rates by trial size

Exclusion criteria by trial size are shown in table 5. Exclusion patterns varied slightly by trial size, with direct exclusion most common in medium-sized trials (36.0%), followed by small (34.6%) and large trials (30.3%). Consent capacity exclusions without assessment were slightly higher in large trials (19.2%) compared with small (15.6%) and medium trials (16.9%). Diagnostic-label exclusions were slightly more frequent in small trials (15.2%) compared with medium (11.6%) and large trials (12.0%). However, explicit exclusion of people with intellectual disability was most frequent in large (6.7%) and small trials (5.3%), compared with medium trials (2.5%). Indirect exclusion was highest in medium trials (33.5%), driven by English language proficiency (19.4%) and access to technology (6.6%). Indirect exclusion was similar in small (20.2%) and large trials (20.7%). Large trials had the highest proportion of trials with no exclusion codes (60.1%), while medium trials had the lowest (47.1%).

**Table 5:** Clinical trials by intervention type

	Drug	Prevention
	**n (%**)	**n (%**)
**Direct exclusion**	32 (42.1)	40 (33.1)
Consent capacity (any code)	19 (25.0)	20 (16.5)
Assessment	1 (1.3)	0 (0.0)
No assessment	19 (25.0)	20 (16.5)
Cognitive capacity (any code)	5 (6.6)	18 (14.9)
Assessment	1 (1.3)	2 (1.7)
No assessment	4 (5.3)	16 (13.2)
Cognitive capacity to engage in study procedures (any code)	7 (9.2)	5 (4.1)
Assessment	0 (0.0)	1 (0.8)
No assessment	7 (9.2)	4 (3.3)
Diagnosis	14 (18.4)	15 (12.4)
Developmental disability	1 (1.3)	3 (2.5)
Intellectual disability	2 (2.6)	7 (5.8)
Other	13 (17.1)	6 (5.0)
**Indirect exclusion**	25 (32.9)	29 (24.0)
Access to technology	0 (0.0)	10 (8.3)
Ability to use technology	1 (1.3)	0 (0.0)
Ability to read and write in English	6 (7.9)	12 (9.9)
Research staff discretion (any code)	19 (25.0)	10 (8.3)
Ability to complete study procedures	4 (5.3)	1 (0.8)
Health	9 (11.8)	7 (5.8)
Potential to confound findings	6 (7.9)	3 (2.5)
Safety	5 (6.6)	1 (0.8)
Other	9 (11.8)	4 (3.3)
**No exclusion codes**	30 (39.5)	64 (52.9)
Total number of trials	76	121

## Discussion

Among 685 Australian government-funded interventional clinical trials, almost half included at least one exclusion criterion with the potential to disproportionately affect adults with intellectual disability. Direct exclusions were more common than indirect exclusions and were most frequently related to consent capacity, applied without specifying how capacity would be assessed. Exclusion based on diagnostic labels also contributed to direct exclusion, with 4.8% of trials excluding people with intellectual disability. Indirect exclusions most often related to English proficiency and staff discretion. Patterns varied by health condition, intervention type and trial size: cancer and drug trials demonstrated the highest frequency of exclusion criteria, while large trials had the highest proportion of protocols without exclusion criteria and the highest rate of explicit exclusion of people with intellectual disability. These patterns indicate that despite many trials lacking exclusionary criteria, a substantial proportion continue to use eligibility practices that may limit participation by adults with intellectual disability.

Across all interventions, 47% of trials included at least one direct or indirect exclusion criterion. Among drug trials, the proportion of Australian drug trials with at least one exclusion code (60.5%) was lower than that reported for NIH-funded drug trials (74.6%)^18^ suggesting that exclusions could be observed globally. Cancer trials had particularly high rates of exclusion, which may reflect greater treatment complexity, safety monitoring, treatment burden and consent-related issues. Exclusion of cognitive impairment occurred in 10.1% of Australian trials, which falls within the broad international range reported across interventional studies. For example, exclusions related to cognitive impairment have been documented in up to 48% of US interventional trials,^4^ whereas considerably lower rates have been reported in more narrowly defined samples such as NIH-funded drug trials (4%).^18^ Exclusions based on consent capacity in our study (17.4%) were comparable to published estimates which span from approximately 8% in critical care trials^17^ to 33% in cancer trials,^20^ and 17.7% across US interventional trials.^4^ As in these international datasets, most consent-related exclusions in Australian trials did not specify any method for assessing capacity. These exclusions may reflect perceived clinical or consent-related risk, but they may also limit access to evidence on the safety and effectiveness of interventions for adults with intellectual disability. When focusing on drug trials, the prevalence of consent-based exclusion in Australian drug trials (25%) was closely aligned with NIH-funded drug trials (25.4%),^18^ and in both contexts these exclusions were almost universally applied without assessment.

Exclusion based on diagnostic labels showed variation when compared with international evidence. The proportion of Australian trials explicitly excluding intellectual disability (4.8% overall and up to 8.2% in mental health trials) was higher than rates reported in comparable international analyses. For example, in NIH-funded drug trials, only 1.6% excluded intellectual disability.^18^ Exclusion of other diagnostic groups (ie, psychiatric diagnosis as per DSM-V) was more common in our sample (8.5% overall and up to 17.1% in drug trials) than in NIH-funded drug trials, where 3.2% excluded other diagnoses.^18^ International studies using broader diagnostic labels have reported higher rates of exclusion, such as behavioural and psychiatric disorders exclusions in 21.9% of US interventional trials.^26^

Indirect exclusion was less frequent in our sample than reported in previous studies. Earlier Australian work found that 21% of trials excluded participants with limited English proficiency,^14^ compared with 12.3% in our dataset. International estimates similarly show substantial variation, with English-language requirements present in 18.9% of US interventional trials and 28.9% among federally funded trials.^27^ Staff discretion codes were less common in our sample (9.2% overall; 25% in drug trials) than in NIH-funded drug trials, where nearly half included discretionary exclusion criteria.^18^ Technology-related exclusion ranged from 0% in drug trials to 8.3% in prevention trials, compared with 9.7% in NIH-funded drug trials.^18^ These technology-related criteria may represent an emerging barrier as digital tools become more integrated into trial procedures.^28^ Together, these comparisons indicate that Australian trials share many of the exclusion patterns reported internationally but also reveal areas where exclusions may be more frequent or operate differently, particularly in relation to exclusions based on diagnostic labels.

Our findings indicate that many Australian government-funded trials remain grounded in eligibility practices that risk systemically excluding adults with intellectual disability, despite national ethics guidance emphasising individualised consent assessment and supported decision-making.^21^ This has several implications for trialists, ethics committees and policymakers responsible for ensuring equitable public benefit from publicly funded research.

First, exclusions based on diagnostic labels require explicit justification. Explicit exclusion of intellectual disability represents an absolute barrier to participation and these exclusions rarely have clear methodological or safety justifications.^4 20^ Exclusions based on diagnostic labels risk perpetuating evidence gaps for a population already facing profound health inequities. Ethics guidance in Australia states that exclusion must not be based on disability alone,^21^ yet our findings suggest that recent trials may still default to diagnosis-linked exclusions without articulating why intellectual disability would compromise safety or validity. Stronger requirements for justification that are embedded within funding calls, ethics review and reporting standards could help trialists to replace categorical diagnostic exclusions with condition-specific, proportionate, evidence-based criteria.^2^

Second, exclusion based on assumed or unspecified consent capacity conflicts with ethical guidance requiring individualised assessment.^21^ Many trials excluded people who were ‘unable to provide consent’ without specifying how capacity would be evaluated. Evidence shows that adults with intellectual disability can often provide informed consent when provided with accessible materials, decision aids or extended discussion.^24 29^ Incorporating supported decision-making processes, including adapted consent materials, would help ensure that consent capacity is assessed rather than presumed and reduce unnecessary exclusion.^30^ Importantly, how supported decision-making works within the Australian research setting is not well explored^31^ but nonetheless encouraged in research ethics guidelines.^21^

Third, indirect exclusion criteria related to language proficiency, technology requirements and investigator discretion are highly modifiable barriers. Such criteria often reflect convenience rather than necessity. Investigator discretion clauses, which appeared in nearly one in ten trials, also merit closer scrutiny. Prior work shows that discretionary exclusions disproportionately disadvantage individuals with psychiatric, cognitive or communication disabilities, particularly when the basis for discretion is subjective or undefined.^16 17^ Replacing open-ended discretion with structured, transparent criteria would improve fairness and reduce the risk of arbitrary exclusion.

Fourth, funders and sponsors of large public trials can play a central role in modelling and enforcing inclusive practice. Large trials in our sample more frequently lacked exclusion criteria, suggesting that scale and infrastructure may enable more inclusive protocols. Inclusive design often requires additional time, resources and staff training which are all factors that may be more readily supported in larger studies.^19 8^ Although exclusions may be more justifiable in early-phase safety studies, routine exclusion of people with intellectual disability from early-phase trials may contribute to their ongoing under-representation across the research pipeline.

Together, these considerations point to the need for more transparent, evidence-based exclusion criteria and stronger support for inclusive trial design, so that adults with intellectual disability are not systematically excluded from publicly funded trials. Australia’s health and medical research strategy highlights equity in trials as a core priority,^10^ but operationalising this vision will require concrete expectations for inclusive eligibility, capacity assessment, accessible materials and recruitment monitoring.

A strength of this study is its broad scope. Our use of an established coding framework^18^ enabled comparison with international data and systematic differentiation between direct and indirect exclusion criteria in a large sample of government-funded interventional trials across multiple conditions, interventions and trial sizes. Focusing on government-funded trials strengthens the policy relevance of the findings, as these trials are intended to deliver public benefit and are expected to align with national priorities for equity and inclusion.^10^ The large sample provides substantial coverage of trials during the study period. While it is possible that some large international trials recruiting in Australia but registered only on other platforms (eg, ClinicalTrials.gov) were not captured, the breadth of the ANZCTR data set likely minimises the impact of any such omissions on overall patterns.

However, several limitations should be noted. First, exclusion criteria listed in registry records do not necessarily reflect recruitment practices or actual enrolment. The ANZCTR fields used in this analysis were mandatory, but the clarity and specificity of the free-text entries varied across records. Some exclusions may not be implemented in practice, while other unreported barriers may arise during recruitment. Second, we did not analyse inclusion criteria or assess the relationship between exclusions and the objectives of the trials. A fuller assessment of whether exclusions are justified would require protocol-level review of trial objectives and scientific rationale and could be future work. Third, the presence of no exclusion criteria in a trial record cannot be interpreted as evidence of practical inclusion and this study does not assess whether the lack of exclusion criteria translates to real inclusion. In addition, because the study was limited to government-funded trials, the findings may not reflect patterns in industry trials. Findings cannot be generalised to paediatric trials and the exclusion of trials with a minimum age of 55 years further limits the generalisability of the findings to older adults with intellectual disability, who remain an important population for clinical research.

Future work is needed to determine how eligibility criteria translate into actual enrolment and whether adults with intellectual disability are meaningfully included in trials that do not explicitly exclude them. Protocol-level inclusivity does not necessarily guarantee practical inclusion and prior work has shown that even when trials are designed to enrol populations who may lack consent capacity, only a small number of such participants are ultimately recruited.^32^ Understanding this gap will require access to participant-level enrolment data and systematic monitoring of recruitment processes.

In addition, qualitative research with trialists, ethics committees and adults with intellectual disability would provide valuable insight into how exclusion criteria are interpreted and applied in practice and how assumptions about capacity or diagnosis shape decision-making. Such work may also illuminate the broader structural and cultural challenges affecting inclusion in Australian trials. Evidence from the UK suggests that while stakeholders broadly support inclusive research, researchers often lack skills, confidence or resources required to implement inclusive practices effectively^29^ and similar gaps may exist in the Australian context. Exploring these issues could help guide system-level reforms, including workforce development, ethics guidance and policy directives to support more inclusive trial design and conduct. In summary, while many trials were designed without exclusionary criteria, a substantial minority rely on diagnostic labels, unassessed capacity assumptions and participation requirements that may unnecessarily limit participation by adults with intellectual disability and perpetuate evidence gaps.

## Data Availability

No data are available.
