# Supplementary material for: How often do Australian government-funded interventional trials exclude adults with intellectual disability? A retrospective registry analysis of ANZCTR records, 2022–2024
Source: BMJ Open. 2026 Oct 20;16(10):e119053. doi: 10.1136/bmjopen-2026-119053 (PMC13684212; doi:10.1136/bmjopen-2026-119053)
Supplement: Supplementary file 1 [file bmjopen-2026-119053.reviewer_comments.pdf]

PEER REVIEW HISTORY

BMJ Open publishes all reviews undertaken for accepted manuscripts. Reviewers are asked to complete a checklist review form and are provided with free text boxes to elaborate on their assessment. These free text comments are reproduced below.

ARTICLE DETAILS

Title (Provisional)

How often do Australian government-funded interventional trials exclude adults with intellectual disability? A retrospective registry analysis of ANZCTR records, 2022-2024

Authors

Kargodorian, Andrea; Christidis, Rebecca; Downs, Jenny; Leonard, Helen; Trollor, Julian

---

VERSION 1 - REVIEW

**Reviewer**	**1**
**Name**	**Finlayson , Janet**
**Affiliation**	**Glasgow Caledonian University, School of Health and Life Sciences**
**Date**	**13-May-2026**
**COI**	**None**

Whilst this is an interesting study, it only tells half the story. As you have highlighted in your Limitations section, we need to understand this in the context of each study's objectives, and more widely in comparison to the studies that do have intellectual disability as their inclusion criteria. I think if you completed this additional analysis, the findings would be more useful to researchers, advocates and funders.

**Reviewer**	**2**
**Name**	**McDonald, Katherine**
**Affiliation**	**Syracuse University, Falk College**
**Date**	**02-Jun-2026**
**COI**	**None**

This paper leverages recent research conducted in other contexts examining whether adults with intellectual disability are likely to be included in clinical trials or not, applying these frameworks to AUS-government funded research. The research is well-conceived, well-conducted, and well-written. The authors make strong arguments for the merit of this new research, and demonstrate rigor in their approach (e.g., appropriate data source, empirically-informed coding criteria, two independent coders, discussion grounded in the findings, etc.).

I have relatively minor suggestions for improvements to the manuscript. These include:

Given that definitions of clinical trials can vary across contexts, the authors should add a definition of clinical trials and interventional clinical trials.

Add a brief rationale for the exclusion of pediatric-only studies (page 8).

Are there any limitations to the dataset that readers should be aware of (including as specifically relates to the free-text field relied upon for this study--is it a required field? Is the text high-quality in terms of clarity, completeness?).

I encourage the authors to consider risks and risk-levels in the discussion section. Do the findings reveal any patterns that have specific potential health consequences for adults with intellectual disability?

The authors note supported decision-making as a potential tool. They might be interested to know that a recent special collection of manuscripts on supported decision-making in clinical research is available from the Journal of Law, Medicine & Ethics.

Lastly, I encourage the authors to use active voice in their description of the steps they took to conduct the research (i.e., the methods)

**Reviewer**	**3**
**Name**	**Waite, Jane**
**Affiliation**	**Aston University**
**Date**	**24-Jun-2026**
**COI**	**None**

This is a timely and important paper addressing the inclusion of adults with intellectual disability in Australian government-funded clinical trials. The study addresses an evidence gap, uses a large national sample of trial registrations, and applies a clear and systematic approach to coding direct and indirect exclusion criteria. The paper is generally well written and the findings have important implications for research equity and inclusive trial design. I have a small number of comments that I believe would strengthen the paper.

1. Exclusion of older adult trials

The justification for the exclusion of trials with a minimum age of 55 years could be further developed. Whilst the authors note concerns regarding age-related cognitive decline, adults with intellectual disability increasingly live into older age and remain an important population for clinical research. 55 is not particularly old and particular illnesses (e.g. some cancers) are diseases of older age. Excluding this age group limits the scope of the findings and may remove an area where assumptions regarding cognitive capacity are particularly relevant. At minimum, I think the implications of this exclusion should be discussed more explicitly and acknowledged as a limitation (including in the summary of limitations at the start of the paper), including that the findings cannot be generalised to older adults with intellectual disability.

2. Exclusion of early-phase trials

The rationale for excluding Phase 0, Phase 1 and Phase 1/2 trials is understandable, given that exclusions may be more justifiable within early-stage safety studies. However, there is also an argument that exclusion at the earliest stages of intervention development may contribute to longer-term under-representation of people with intellectual disability across the research pipeline. If people with intellectual disability are routinely excluded from early-stage research, interventions may subsequently lack the evidence base required to support inclusion in later-phase studies. It may be important to acknowledge this perspective within the discussion.

3. Coding procedures and reliability

The manuscript states that exclusion criteria were independently coded by two researchers and discrepancies resolved through discussion. However, it is unclear whether all trial records underwent independent double

coding and no inter-rater reliability statistics are reported. Given that the study relies on interpretation of free-text eligibility criteria, reporting agreement statistics (e.g., percentage agreement or kappa values) would strengthen confidence in the coding process.

4. Statistical analysis

The manuscript presents several interesting differences across health conditions, intervention types, and trial sizes. Given the relatively large sample size, I wondered whether inferential analyses were considered to determine whether observed differences were statistically meaningful. I suspect inferential statistics would have been untenable given the number of comparisons, however, if the intention was for the study to remain purely descriptive, it would be helpful for the authors to briefly justify this decision.

Minor comment

Cancer trials appeared to have particularly high rates of both direct and indirect exclusion. This is an interesting finding and I wondered whether the discussion could provide greater consideration of possible explanations, such as treatment complexity, safety concerns, treatment burden, or consent-related issues.

VERSION 1 - AUTHOR RESPONSE

Reviewer 1 – Dr. Janet Finlayson, Glasgow Caledonian University

**Comment**	**Response**
Whilst this is an interesting study, it only tells half the story. As you have highlighted in your Limitations section, we need to understand this in the context of each study's objectives, and more widely in comparison to the studies that do have intellectual disability as their inclusion criteria. I think if you completed this additional analysis, the findings would be more useful to researchers, advocates and funders.	Thank you for this important comment. We agree that assessing whether exclusions are justified by trial objectives is a valuable question. However, this is not a question that can be answered by ANZCTR registry records alone, because determining whether an exclusion was justified requires protocol-level information about each trial’s objectives, intervention risk, and scientific rationale (*please see references:* 1, 2, 3, & 4). Addressing this would constitute a different research question and require a different methodology, so it was beyond the scope of the present registry-based audit. Where clinical trial registries have been used to support this type of research question, it is only to find the relevant subset of trials to review.<br><br>(1) DOI: 10.1001/jama.297.11.1233<br>(2) DOI: 10.1377/hlthaff.2022.00520<br>(3) DOI: 10.1016/j.dhjo.2025.101824<br>(4) DOI: 10.1093/jnci/djae338

	We have revised the Discussion to note this as potential future work. The revised text on **line 388** reads: "**A fuller assessment of whether exclusions are justified would require protocol-level review of trial objectives and scientific rationale and could be future work.**"

Reviewer 2 – Dr. Katherine McDonald, Syracuse University

**Comment**	**Response**
This paper leverages recent research conducted in other contexts examining whether adults with intellectual disability are likely to be included in clinical trials or not, applying these frameworks to AUS-government funded research. The research is well-conceived, well-conducted, and well-written. The authors make strong arguments for the merit of this new research, and demonstrate rigor in their approach (e.g., appropriate data source, empirically-informed coding criteria, two independent coders, discussion grounded in the findings, etc.). I have relatively minor suggestions for improvements to the manuscript. These include:	We thank the reviewer for their positive assessment of the manuscript and for the helpful suggestions provided to the strengthen the paper, which are addressed below.

2.1 Given that definitions of clinical trials can vary across contexts, the authors should add a definition of clinical trials and interventional clinical trials.	Thank you for this comment. We have revised the methods to clarify that clinical trial records were identified using the ANZCTR, which applied WHO-compliant wording for interventional studies.<br><br>The revised Methods sentence on **line 134** reads: "We identified clinical trial records using the advanced search function on the Australian New Zealand Clinical Trials Registry (ANZCTR; accessed at: https://www.anzctr.org.au/TrialSearch.aspx), **which uses WHO-compliant definitions for interventional studies.**
2.2 Add a brief rationale for the exclusion of pediatric-only studies (page 8).	Thank you for this comment. We have added a brief rationale for excluding paediatric-only trials, noting that the study focused on adult participation.<br>The revised Methods sentence on **line 206** reads: "trials with only child participants (maximum age <18 years, n = 57) **were excluded because the audit focused on adult participation in clinical trials;** studies with both child and adult participants **were retained.**"
2.3 Are there any limitations to the dataset that readers should be aware of (including as specifically relates to the free-text field relied upon for this study--is it a required field? Is the text high-quality in terms of clarity, completeness?).	Thank you for this comment. We have clarified in the limitations section that the ANZCTR fields used in this analysis were mandatory, but the clarity and specificity of the free-text entries varied across records.<br><br>The revised Discussion on **line 384** reads: "**The ANZCTR fields used in this analysis were mandatory, but the clarity and specificity of the free-text entries varied across records.**"

Comment	Response
I encourage the authors to consider risks and risk-levels in the discussion section. Do the findings reveal any patterns that have specific potential health consequences for adults with intellectual disability?	Thank you for this comment. We have added a sentence to the discussion noting that the observed exclusions may reflect perceived clinical or consent-related risk. We have kept this cautious because the study did not assess trial protocols or scientific rationale for exclusions.<br><br>The revised Discussion on **line 298** reads: **"These exclusions may reflect perceived clinical or consent-related risk, but they may also limit access to evidence on the safety and effectiveness of interventions for adults with intellectual disability."**
The authors note supported decision-making as a potential tool. They might be interested to know that a recent special collection of manuscripts on supported decision-making in clinical research is available from the Journal of Law, Medicine & Ethics.	Thank you for this helpful suggestion. We have added a recent reference on supported decision-making in clinical research to the Discussion, where supported decision-making is discussed as a potential approach to reduce unnecessary exclusion.
Lastly, I encourage the authors to use active voice in their description of the steps they took to conduct the research (i.e., the methods)	Thank you for noting this. We have revised the Methods section to use more consistent active voice throughout. The following lines in the method have been revised to active voice: 139, 141, 148, 149, 151, 162-164, 175, 191, 195, and 205.

Reviewer 3 – Dr. Jane Waite, Aston University

Comment	Response

This is a timely and important paper addressing the inclusion of adults with intellectual disability in Australian government-funded clinical trials. The study addresses an evidence gap, uses a large national sample of trial registrations, and applies a clear and systematic approach to coding direct and indirect exclusion criteria. The paper is generally well written and the findings have important implications for research equity and inclusive trial design. I	We thank the reviewer for their positive assessment of the manuscript and for the helpful suggestions provided to the strengthen the paper, which are addressed below.

have a small number of comments that I believe would strengthen the paper.	
1. Exclusion of older adult trials<br><br>*The justification for the exclusion of trials with a minimum age of 55 years could be further developed. Whilst the authors note concerns regarding age-related cognitive decline, adults with intellectual disability increasingly live into older age and remain an important population for clinical research. 55 is not particularly old and particular illnesses (e.g. some cancers) are diseases of older age. Excluding this age group limits the scope of the findings and may remove an area where assumptions regarding cognitive capacity are particularly relevant. At minimum, I think the implications of this exclusion should be discussed more explicitly and acknowledged as a limitation (including in the summary of limitations at the start of the paper), including that the findings cannot be generalised to older adults with intellectual disability.*	Thank you for this comment. We agree that older adults with intellectual disability remain an important population for clinical research, and that this age-based exclusion limits the generalisability of the findings. We have added this point to the dot points for “Strengths and limitations of this study” and to the Discussion. Fully addressing whether the excluded trials were primarily targeting age-related cognitive decline would require closer scrutiny of trial aims and was beyond the scope of the present audit.<br><br>The revised limitation on **line 74** now reads: “Data and findings cannot be generalised to children **or older adults** with intellectual disability.”<br><br>The revised Discussion on **line 394** now reads: “Findings cannot be generalised to paediatric trials, **and the exclusion of trials with a minimum age of 55 years further limits the generalisability of the findings to older adults with intellectual disability, who remain an important population for clinical research.”**

2. Exclusion of early-phase trials<br><br>*The rationale for excluding Phase 0, Phase 1 and Phase 1/2 trials is understandable, given that exclusions may be more justifiable within early-stage safety studies. However, there is also an argument that exclusion at the earliest stages of intervention development may contribute to longer-term under-representation of people with intellectual disability across the research pipeline. If*	Thank you for this comment. We have added a brief point in the Discussion noting that, although exclusions may be more justifiable in early-stage safety studies, routine exclusion from early-phase trials may contribute to longer-term under-representation of people with intellectual disability across the research pipeline.<br><br>The revised Discussion on **line 363** now reads: "**Although exclusions may be more**
people with intellectual disability are routinely excluded from early-stage research, interventions may subsequently lack the evidence base required to support inclusion in later-phase studies. It may be important to acknowledge this perspective within the discussion.	**justifiable in early-phase safety studies, routine exclusion of people with intellectual disability from early-phase trials may contribute to their ongoing under-representation across the research pipeline.**"
3. Coding procedures and reliability<br><br>*The manuscript states that exclusion criteria were independently coded by two researchers and discrepancies resolved through discussion. However, it is unclear whether all trial records underwent independent double coding and no inter-rater reliability statistics are reported. Given that the study relies on interpretation of free-text eligibility criteria, reporting agreement statistics (e.g., percentage agreement or kappa values) would strengthen confidence in the coding process.*	Thank you for this comment. We have clarified that all included trial records were independently double coded by two authors before consensus discussion. Inter-rater reliability statistics were not reported because the final codes were resolved through consensus.<br><br>The revised method on **line 180** now reads: "**All included trial records were** coded independently by two authors (AK and RC)…"

4. Statistical analysis<br><br>*The manuscript presents several interesting differences across health conditions, intervention types, and trial sizes. Given the relatively large sample size, I wondered whether inferential analyses were considered to determine whether observed differences were statistically meaningful. I suspect inferential statistics would have been untenable given the number of comparisons, however, if the intention was for the study to remain purely descriptive, it would be helpful for the authors to briefly justify this decision.*	Thank you for this comment. We have revised the Statistical analysis section to clarify that the study was designed to summarise registry data rather than specific hypotheses, and that the subgroup comparisons were exploratory.<br><br>The revised method on **line 195** now reads: "**We used a descriptive approach because the study aimed to summarise registry data, and subgroup comparisons were exploratory rather than hypothesis driven.**"
Minor comment<br><br>*Cancer trials appeared to have particularly high rates of both direct and indirect exclusion. This is an interesting finding and I wondered whether the discussion could provide greater consideration of possible explanations, such as treatment complexity, safety concerns, treatment burden, or consent-related issues.*	Thank you for this suggestion. We have expanded the Discussion to note that the higher exclusion rates in cancer trials may reflect treatment complexity, safety monitoring, treatment burden, and consent-related issues.<br><br>The revised Discussion on **line 287** now reads: "**Cancer trials had particularly high rates of exclusion, which may reflect greater treatment complexity, safety monitoring, treatment burden, and consent-related issues.**"

VERSION 2 - REVIEW

**Reviewer**	**2**
**Name**	**McDonald, Katherine**
**Affiliation**	**Syracuse University, Falk College**
**Date**	**31-Jul-2026**
**COI**	

Thank you for the revisions to this manuscript.
